# Nanoparticles Loaded with the BET Inhibitor JQ1 Block the Growth of Triple Negative Breast Cancer Cells In Vitro and In Vivo

**DOI:** 10.3390/cancers12010091

**Published:** 2019-12-30

**Authors:** Valentina Maggisano, Marilena Celano, Rocco Malivindi, Ines Barone, Donato Cosco, Catia Mio, Chiara Mignogna, Salvatore Panza, Giuseppe Damante, Massimo Fresta, Sebastiano Andò, Diego Russo, Stefania Catalano, Stefania Bulotta

**Affiliations:** 1 Department of Health Sciences, “Magna Graecia” University of Catanzaro, 88100 Catanzaro, Italy; vmaggisano@unicz.it (V.M.); celano@unicz.it (M.C.); donatocosco@unicz.it (D.C.); fresta@unicz.it (M.F.); bulotta@unicz.it (S.B.); 2 Department of Pharmacy, Health and Nutritional Sciences, University of Calabria, 87036 Cosenza, Italy; rocco.malivindi@unical.it (R.M.); ines.barone@unical.it (I.B.); sasapanza@libero.it (S.P.); sebastiano.ando@unical.it (S.A.); 3 Department of Medical Area, University of Udine, 33100 Udine, Italy; mio.catia@spes.uniud.it (C.M.); giuseppe.damante@uniud.it (G.D.); 4 Interdepartmental Service Center, “Magna Graecia” University of Catanzaro, 88100 Catanzaro, Italy; mignogna@unicz.it

**Keywords:** triple negative breast cancer, targeted therapy, nanoparticles, BET inhibitors, JQ1

## Abstract

Inhibition of bromo-and extra-terminal domain (BET) proteins, epigenetic regulators of genes involved in cell viability, has been efficiently tested in preclinical models of triple negative breast cancer (TNBC). However, the use of the selective BET-inhibitor JQ1 on humans is limited by its very short half-life. Herein, we developed, characterized and tested a novel formulation of nanoparticles containing JQ1 (N-JQ1) against TNBC in vitro and in vivo. N-JQ1, prepared using the nanoprecipitation method of preformedpoly-lactid-co-glycolic acid in an aqueous solution containing JQ1 and poloxamer-188 as a stabilizer, presented a high physico-chemical stability. Treatment of MDA-MB 157 and MDA-MB 231 TNBC cells with N-JQ1 determined a significant decrease in cell viability, adhesion and migration. Intra-peritoneal administration (5 days/week for two weeks) of N-JQ1 in nude mice hosting a xenograft TNBC after flank injection of MDA-MB-231 cells determined a great reduction in the growth and vascularity of the neoplasm. Moreover, the treatment resulted in a minimal infiltration of nearby tissues. Finally, the encapsulation of JQ1 in nanoparticles improved the anticancer efficacy of this epigenetic compound against TNBC in vitro and in vivo, opening the way to test it in the treatment of TNBC.

## 1. Introduction

Breast cancer is the most common female malignant tumor around the world, accounting for approximately 30% of all new cancer diagnoses in women [1,2]. The current classification that governs the therapeutic approach depends mainly on the expression of the estrogen receptor (ER) and progesterone receptor (PR), as well as on the amplification status of human epidermal growth factor receptor 2 (HER2). The lack of these receptors characterizes triple negative breast cancer (TNBC), which accounts for 12% to 17% of all breast malignant tumors. This subtype is known to affect younger women and is associated with a high frequency of distant metastasis and poor prognosis [3]. Only early-stage TNBC is chemotherapy-sensitive [3], thus the search for more effective treatments is still an open challenge.

A novel and promising approach is based on the use of compounds able to target the epigenome of breast cancer cells. In particular, among the molecules that inhibit the transcription of growth-promoting genes by acting on the “readers” of the acetylated histone marks, the bromo-and extra-terminal domain (BET) inhibitor JQ1 ((S)-tertbutyl2-(4-(4-chlorophenyl)-2,3,9-trimethyl-6H-thieno(3,2-f)(1,2,4)triazolo(4,3-a)(1,4)diazepin-6-yl)acetate) has shown a strong anti-proliferative action against TNBC cell lines and xenografts [4,5,6,7]. JQ1 acts by competing with acetylated histone residues, thus removing BET proteins from their chromatin targets. It has been tested for its anticancer effects in both solid and hematological preclinical models of malignancies [4,8,9].

The potential use of JQ1 on humans, however, is limited by its very short half-life, ~1 h [10], prompting the need for particulate carriers or specific conjugates to deliver this compound to its targets. For this purpose, the nanoencapsulation of a drug in a biocompatible polymeric colloidal system is a pharmaceutical approach widely used in the last decades to increase the pharmacological efficacy of the active compound, modulate its pharmacokinetic profile and decrease its side effects and the administration times [11]. In order to obtain nanosystems able to deliver various active compounds against cancer cells, among the various polymeric biomaterials, we and others have used the poly-lactid-co-glycolic acid (PLGA) polymer, which is approved by the U.S. FDA and EMA for pharmaceutical application [12,13,14].

In this work, we have developed and characterized the properties of PLGA nanoparticles containing JQ1 (N-JQ1) and evaluated the anticancer efficacy of this new formulation against TNBC cells in vitro and in vivo.

## 2. Results

### 2.1. Physico-Chemical Characterization of the Nanosystems

The first step of this investigation was focused on the development and evaluation of the physico-chemical properties of JQ1-loaded PLGA nanoparticles. The polymeric nanosystems were characterized by a mean diameter of 130–150 nm, a good size distribution and a negative surface charge (Table 1). The encapsulation of JQ1 did not compromise the aforesaid parameters up to a drug concentration of 0.5 mg/mL, confirming the peculiar properties of PLGA as a useful biomaterial in the development of colloidal systems for the delivery of lipophilic active compounds [15]. The obtained results are in agreement with those previously described by our research team when other lipophilic compounds have been entrapped within PLGA nanoparticles [16,17,18].

**Figure 1:**
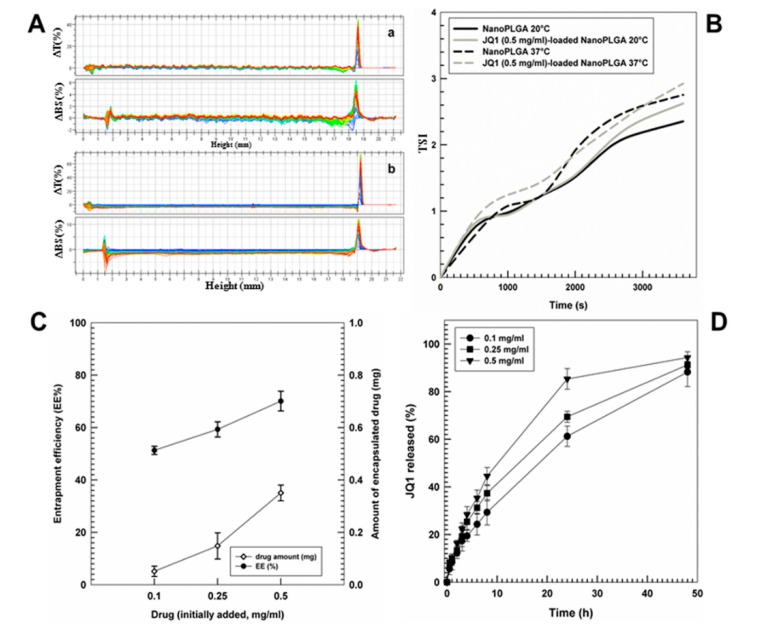
Evaluation of the physical stability of the various nanoformulations. (**A**) Transmittance (ΔT) and backscattering (ΔBS) profiles of (**a**) empty PLGA nanoparticles and (**b**) nanosystems prepared with JQ1 (0.5 mg/mL) using Turbiscan Lab. Results representative of three independent experiments are shown. (**B**) Turbiscan Stability Index (TSI) profiles of PLGA nanoparticles as empty formulation or prepared with JQ1 (0.5 mg/mL) as a function of time and temperature. (**C**) Entrapment efficiency of JQ1 in PLGA nanoparticles as a function of the drug concentration used. (**D**) Release profile of JQ1 from PLGA nanoparticles as a function of the entrapped drug concentration and incubation time. Values represent the mean of three different experiments ± SD.

The evaluation of backscattering and transmittance profiles of the various systems demonstrated a strong stability of nanoparticles (Figure 1A). Moreover, the temperature did not compromise the aforesaid parameters, demonstrating a great stability of the systems at 37 °C (Figure 1B). In fact, the Turbiscan Stability Index (TSI) profiles of the formulations were characterized by the absence of significant variations over the time, confirming the absence of sediment, flocculation or creaming. The evaluation of the entrapment efficiency showed a proportional improvement of the retention rate of the active compound when the concentration of JQ1 added during the preparation steps of nanosystems was increased (Figure 1C). Moreover, the drug leakage from the polymeric colloidal structure was prolonged and influenced by the concentration of the active compound; namely, a full leakage of the entrapped compound was obtained after 48 h (Figure 1D).

### 2.2. Effects of JQ1-Loaded Nanoparticles on Growth, Migration and Adhesion of TNBC Cells In Vitro

**Figure 2:**
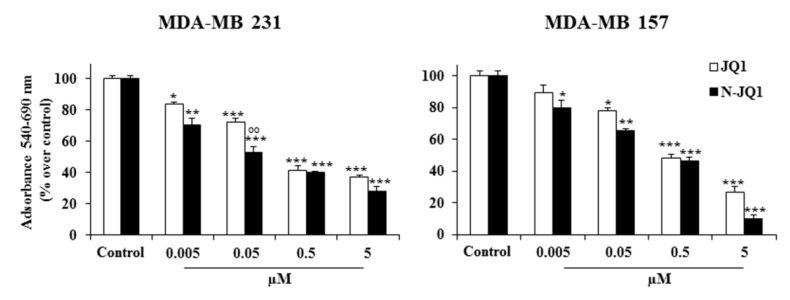
Effects on TNBC cell viability. MDA-MB 231 and MDA-MB 157 cells were treated with JQ1 diluted in PBS + 5% PEG 400 + 5% TWEEN (JQ1) or encapsulated in nanoparticles (N-JQ1). Effects on viability were analyzed by MTT assay. Each experiment was performed in triplicate and values are expressed in % over Control, as means ± SD. Statistical analysis was performed using the Tukey–Kramer multiple comparisons test. * *p* < 0.05, ** *p* < 0.01, *** *p* < 0.001 vs. Control; °° *p* < 0.01 vs. JQ1. Control, cells treated with JQ1 vehicle (white bars) or empty nanoparticles (black bars).

Treatment for 48 h with the biocompatible nanoformulation containing JQ1 (N-JQ1) at various concentrations (0.005, 0.05, 0.5 and 5 µM) determined a significant reduction in viability of both MDA-MB 231 and MDA-MB 157 cells (Figure 2). In particular, a decrease of approximately 50% vs. untreated cells was observed at the 0.05 µM concentration of N-JQ1 in MDA-MB 231 cells, with a significantly stronger effect compared with JQ1. Similar effects were detected in MDA-MB 157 cells, but with an EC_50_ of 0.5 µM (Figure 2).

**Figure 3:**
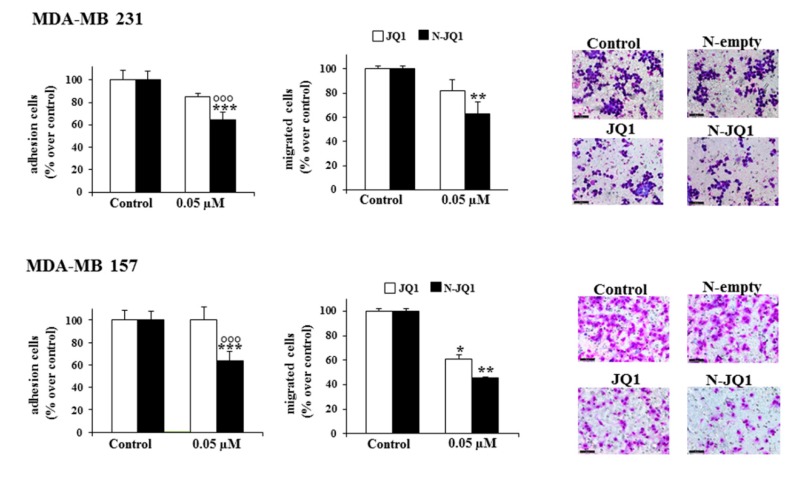
Effects of N-JQ1 on adhesion and migration properties of TNBC. MDA-MB 231 and MD-MB 157 cells were prepared for adhesion and migration assays as indicated in methods. Each experiment was performed in triplicate and values are expressed in % over Control, as means ± SD. Statistical analysis was performed using the one-way ANOVA test. * *p* < 0.05, ** *p* < 0.01, *** *p* < 0.001 vs. Control; °°° *p* < 0.001 vs. JQ1. Representative images of stained cells after migration assays are shown. Control, cells treated with JQ1 vehicle (white bars) or empty nanoparticles (black bars). Scale bar: 50 µm.

Treatment with N-JQ1 0.05 µM also induced a significant reduction in adhesion and migration of MDA-MB 231 (~40% and ~50%, respectively) and MDA-MB 157 (~40% and ~70%, respectively) cells compared to the vehicle treatment (Figure 3). Again, in MDA-MB 231 cells, the effects of N-JQ1 were stronger than those of JQ1 (Figure 3).

Altogether, these preliminary data in vitro demonstrated that the obtained nanoformulation preserves and increases the antitumor efficacy of JQ1, and supported our choice to continue the study using an in vivo experimental model.

### 2.3. Effects of JQ1-Loaded Nanoparticles on Breast Cancer Xenograft In Vivo

To check the anticancer activity of N-JQ1 against TNBC in vivo, we administered N-JQ1 in athymic nude mice hosting xenograft tumors obtained by intrascapular injection of MDA-MB 231 cells in 0.1 mL of Matrigel. Another group of animals was treated with JQ1 diluted in PBS + 5% PEG 400 + 5% TWEEN 80% (JQ1). As controls, we used animals treated with unloaded nanoparticles (N-empty) or PBS + 5% PEG 400 + 5% TWEEN 80 (Control). These treatments were well tolerated since no modifications of body weight or of food and water consumption as well as no evidence of reduced motor function were detected.

**Figure 4:**
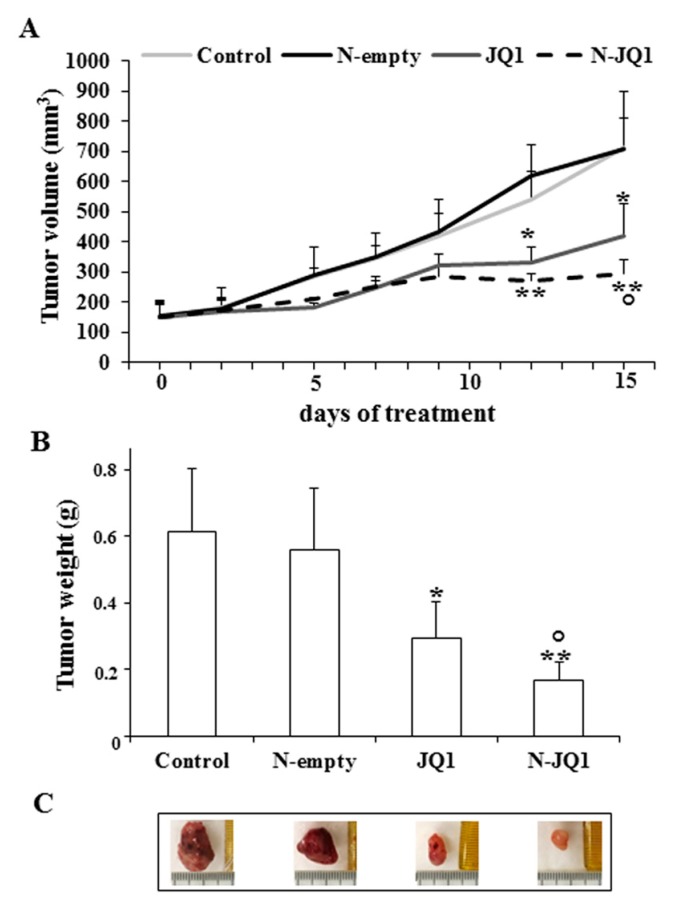
Effects of N-JQ1 treatment on xenograft tumor growth obtained after injection of MDA-MB 231 cells into the intrascapular region of female athymic nude mice. JQ1 (20 mg/kg) diluted in PBS + 5% PEG 400 + 5% TWEEN 80 (JQ1), nanoparticles containing JQ1 20 mg/Kg (N-JQ1), unloaded nanoparticles (N-empty) and PBS + 5% PEG 400 + 5% TWEEN 80 (Control) were administered by intraperitoneal injection for 15 days (5 days a week) as described in Methods. (**A**) tumor volume during the treatment; (**B**) tumor weight at sacrifice. Values are expressed as the mean of animal groups ± SD. Statistical analysis was performed by using the one-way ANOVA test. * *p* < 0.05, ** *p* < 0.01 vs. Control or empty nanoparticles; ° *p* < 0.05 vs. JQ1. (**C**) images of representative individual tumors for each group of mice.

As shown in Figure 4A, tumor growth was decreased in the mice treated with JQ1. Interestingly, a more significant regression of tumor growth was observed in the mice treated with N-JQ1 (Figure 4A). At the end of treatments, tumors of the mice treated with N-JQ1 presented lower weights and sizes than the group of controls or the one treated with the empty nanoparticles (N-empty). A significant difference was also noted between the groups treated with N-JQ1 versus those receiving JQ1 (Figure 4B,C).

**Figure 5:**
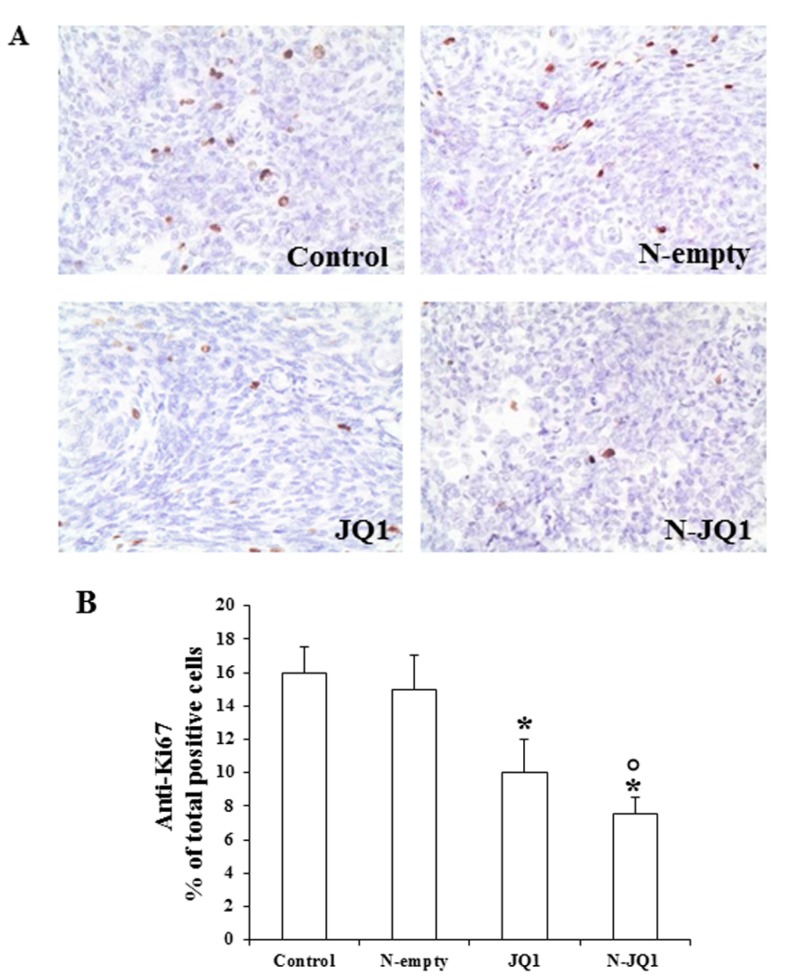
(**A**) A representative image of immunostaining for Ki67 in tumors of each group of animals, treated as described in Methods (200× magnification). (**B**) The percentage of positive over total stained cells for Ki67 in tumors slices are shown in bar graphs.JQ1, JQ1 (20 mg/kg) diluted in PBS + 5% PEG 400 + 5% TWEEN; N-JQ1, nanoparticles containing JQ1 20 mg/Kg; N-empty, unloaded nanoparticles; Control, untreated mice. Values are expressed as means ± SD. * *p* < 0.05 vs. Control; ° *p* < 0.05vs. JQ1.

In accordance with the reduced proliferation, immunohistochemical analysis of the tumors showed a significant reduction of the levels of Ki67 in the tumors treated with N-JQ1 (Figure 5).

**Figure 6:**
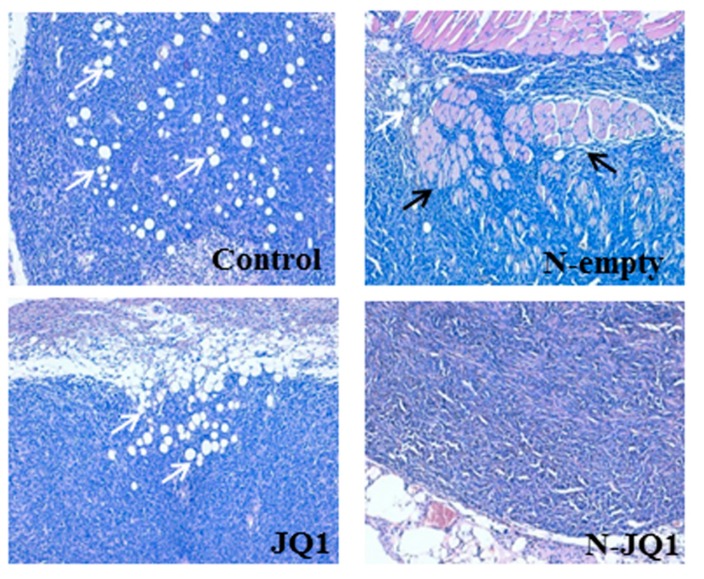
Tumor infiltration of tumor-surrounding tissues. Analyses of tumor tissues by hematoxylin and eosin staining of the four groups of animals after treatment with JQ1 (20 mg/kg) diluted in PBS + 5% PEG 400 + 5% TWEEN (JQ1), unloaded nanoparticles (N-empty) or nanoparticles containing JQ1 20 mg/Kg (N-JQ1). Black and white arrows indicate muscle and fat infiltrations, respectively. Magnification 100×.

In addition, we noted that the tumors of the control groups, as well as those treated with empty nanoparticles were particularly aggressive, exhibiting a large infiltration of the surrounding adipose and muscle tissues, which was reduced in the animals treated with JQ1 or N-JQ1 (Figure 6). However, no signs of metastases in liver, kidney or lungs were observed at the sacrifice of the animals.

**Figure 7:**
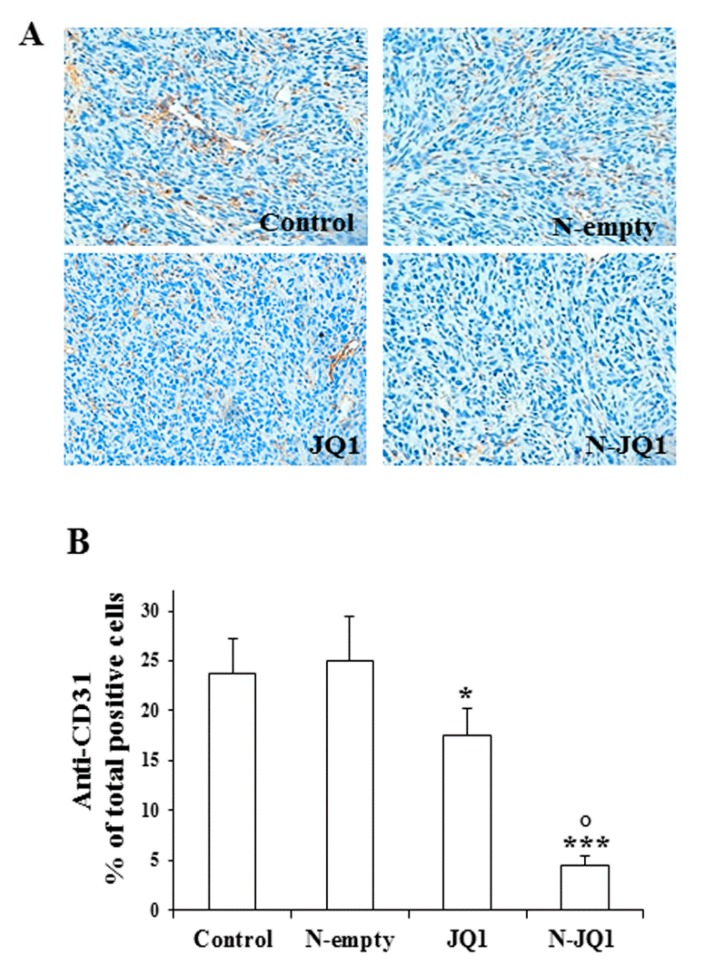
Immunohistochemical analysis of CD31 expression. (**A**) Representative images of immunostaining for CD31 in tumors of the four groups of animals treated as described in Methods (200× magnification). (**B**) The percentage of total positive cells for CD31 tumors are shown in bar graphs. JQ1, JQ1 (20 mg/kg) diluted in PBS + 5% PEG 400 + 5% TWEEN; N-JQ1, nanoparticles containing JQ1 20 mg/Kg; N-empty, unloaded nanoparticles; Control, untreated mice. Values are expressed as means ± SD. * *p* < 0.05, *** *p* < 0.001 vs. control; ° *p* < 0.05 vs. JQ1.

Moreover, a decreased rate of vascularization was also observed in the tumors of N-JQ1 group vs. the controls, as revealed by CD31 immunostaining of the vascular compartment of tumors (Figure 7).

Histological examination of liver, kidney and lungs showed normal tissue morphology indicating a lack of toxicity at the dose of JQ1 used.

**Figure 8:**
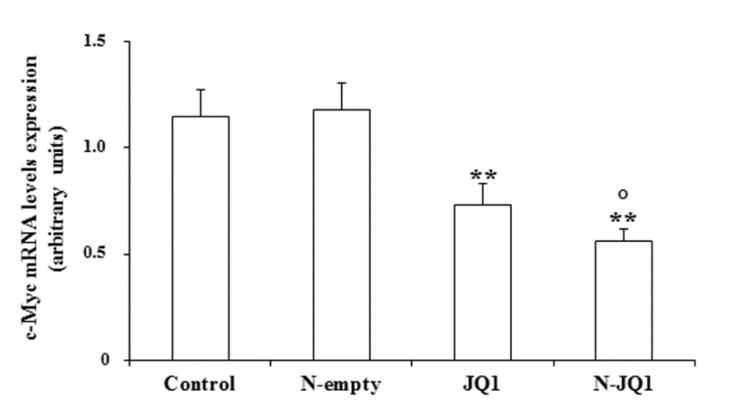
c-Myc mRNA levels in tumor tissues. Control, untreated mice JQ1; N-empty, unloaded nanoparticles; JQ1 (20 mg/kg) diluted in PBS + 5% PEG 400 + 5% TWEEN; N-JQ1, nanoparticles containing JQ1 20 mg/Kg. Values are expressed as means ± SD. * *p* < 0.05 vs. control, ** *p* < 0.01 vs. N-empty; ° *p* < 0.05 vs. JQ1.

Since a decrease in c-Myc expression is a major target of BET inhibitor action, we analyzed c-Myc mRNA levels in tumor tissues after N-JQ1 treatment. We found that N-JQ1 significantly reduced the expression of *c-Myc* (Figure 8).

## 3. Discussion

A more effective treatment of the patients with breast cancer unresponsive to chemotherapy after surgical treatment represents a still unresolved clinical challenge. While good results are being obtained with the endocrine therapy in patients with tumors expressing ER and/or PR, as well as HER2-targeted therapies used against tumors with HER2 amplification, no efficacious drugs are available for the TNBC, the most aggressive breast neoplasm marked by early relapse and a predominance of distant metastases [3,19].

Many approaches are currently under investigation, including the search for molecular targets, potential candidates for selective treatments [20]. For this purpose, targeting histone activity in regulating gene expression by acting on epigenome “readers” is a promising approach for the treatment of various malignancies, including TNBC [9,21,22]. In particular, JQ1 is a first-in-class selective BET bromodomain inhibitor [23], which acts by competitively binding with high affinity to the acetyl-lysine hydrophobic pocket of BRD4 [24,25]. Thus, it displaces BET bromodomains from chromatin, interfering with BRD4 function and leading to growth arrest and promotion of apoptosis in tumor cells [26].

A major problem, which may limit the use of JQ1 in humans, is represented by its very short half-life [10]. In this work, to overcome the rapid degradation of JQ1, we prepared, characterized and then tested in vitro and in vivo in preclinical models of TNBC a novel formulation of nanodevices loaded with JQ1, obtaining a compound with high stability in physiological solutions. We found that treatment of TNBC cells with N-JQ1 determined a significant decrease of cell viability and a reduction of cell adhesion and migration. These results prompted us to evaluate the effects of this novel formulation in vivo. Intra-peritoneal (i.p.) administration of N-JQ1 in nude mice hosting a xenograft cancer obtained by flank injection of MDA-MB 231 cells, determined a great reduction in the growth of the neoplasm compared with tumors in mice receiving empty nanoparticles, associated with a minimal infiltration of nearby tissues and a reduced vascularity of the tumors. The latter effects are particularly relevant, considering the aggressive behavior of these neoplasms. In addition, no toxic effects were observed at the doses used.

A well-recognized approach able to prolong the plasmatic half-life of a colloidal system is based on the coating of its surface by means of hydrophilic derivatives (as in the case of PEG) with the aim of decreasing its identification and elimination [27]. However, the multiple administration of PEG-coated systems determines the appearance of an accelerated blood clearance (the so-called ABC phenomenon) [28,29]. For this reason, the PLGA nanoparticles proposed in our work were developed by using the poloxamer 188 as a stabilizer because it can confer long circulating properties to the particles, avoiding the use of PEG [30,31]. It is important to evidence that DMSO (alone or in combination with 2-hydroxypropyl-β-cyclodextrin) and a surfactant-based system made up of Tween 80 and PEG, which have been used to solubilize the drug (“free JQ1”) in the in vitro and in vivo experiments [32,33], are mixtures associated with several adverse events on humans. Thus, their use is discouraged by the FDA reports. To the best of our knowledge, no similar drawbacks have been reported by using the biomaterials of our PLGA-basednanoparticles. The formulation used to deliver JQ1 in the present study, in addition to its high physical stability, is biocompatible and is made up of components already approved for human applications [34,35,36].

Various other formulations have been developed in order to entrap JQ1; bisphosphonate-functionalized hydroxyapatite-, phospholipid- and chitosan-based nanostructures containing the active compound have been characterized and proposed for the treatment of various diseases, including osteosarcoma, glioma and liver fibrosis, respectively [37,38,39]. Biomimetic nanoclusters made up of the components of the platelet membrane and polyamidoamine–polyvalerolactone–COOH (PAMAM–PVL–COOH) have also been described as a potential endothelium-protective anti-restenotic system, improving the peculiar pharmacological properties of JQ1 [40]. Other potential advantages of our biocompatible systems are (i) to protect the drug from the chemical, physical and enzymatic degradation;(ii) to localize high amounts of compound in the cells as “cargo”, modulating its cell availability as a function of the leakage profile from the carriers; and (iii) to contrast the mechanisms of cell resistance by peculiar properties of specific components of the formulation (for example the inhibition of PgP efflux pumps exerted by various poloxamerderivatives). Furthermore, the possibility of co-encapsulation of other active compounds offered by this kind of nanoparticles may represent an additional advancement of the anticancer features of this nanoformulation. Indeed, the recent direction of the research of anticancer use of BET inhibitors suggests the use of combinations with other chemotherapeutics, including associations inside the same delivery biosystems [9,22,38,41,42].

Findings reported herein are very promising to propose the in vivo use of JQ1 in TNBC patients. Obviously, the biodistribution and pharmacokinetic profiles of the proposed nanoformulation need further investigation in order to understand the tissue distribution of this nanomedicine and to define the best administration regimen.

## 4. Methods

### 4.1. Preparation and Characterization of JQ1-Loaded PLGA Nanoparticles

PLGA nanoparticles containing JQ1 (0.1–1 mg/mL) were prepared as previously described [16]. Briefly, 2 mL of acetone containing various amounts of drug and PLGA (0.6% w/v) (Sigma Aldrich, Milan, Italy) were mixed with an aqueous solution (5 mL) enriched with poloxamer 188 (1% w/v) (Pluronic^®^ PE 6800; BASF, Aktiengesellschaft, Ludwigshafen, Germany) by an Ultraturrax (Ultraturrax T25, IKA^®^Werke) at 24,000 rpm for 1 min. Successively, the suspension was purified and characterized as previously reported [16]. The physico-chemical properties of PLGA nanoparticles were investigated by photon correlation spectroscopy in order to evaluate the mean diameter, size distribution and surface charge [43]. The physical stability of nanoparticles was evaluated as a function of the temperature by using the Turbiscan Lab^®^ analyzer according to procedures described elsewhere [44]. The amount of JQ1 retained by the PLGA nanoparticles was evaluated by a spectrophotometric method; briefly, the systems were centrifuged (70,000 rpm, 4 °C for 1 h, Optima TL Ultracentrifuge, Beckman Coulter, Milan, Italy) and the pellet dissolved in acetone. The solvent was then removed under a nitrogen flux and JQ1 solubilized in ethanol and analyzed by a spectrophotometer at the wavelength of 256 nm. The entrapment efficiency (EE%) of JQ1 was evaluated according to the following equation: EE% = De/Da × 100, where De is the amount of entrapped JQ1 and Da is the amount of active compound used during the preparation of the colloidal formulations. The release profile of JQ1 from the polymeric systems was investigated by the dialysis method [45].

### 4.2. TNBC Cell Lines and Proliferation Assay

MDA-MB 231 and MDA-MB 157, human breast cancer epithelial cells widely used as models of TNBC for characteristics of growth and progression, were purchased from the American Type Culture Collection (Manassas, VI, USA), and cultured as previously reported [21]. Cell proliferation was evaluated by MTT assay [46], performed in 96-well plates containing MDA-MB 231 and MDA-MB 157 cells seeded at a concentration of 5 × 10^3^ and 3.5 × 10^3^/well, respectively, and maintained in medium with 10% foetal bovine serum (FBS). After treatment with JQ1 diluted in DMSO as vehicle, empty nanoparticles or nanoparticles containing different concentrations of JQ1 (N-JQ1) for 48 h, the crystals of formazan were quantified with a microplate spectrophotometer (Thermo Fisher Multiskan FC, Thermo Fisher Scientific Inc., Waltham, MA, USA) at a wavelength of 540 nm and a reference wavelength of 690 nm. Results are expressed as percentages over untreated cells (JQ1 vehicle or empty nanoparticles) indicated as Control.

### 4.3. Adhesion and Migration Assay

Adhesion and migration assays were performed as previously described [47]. The cells were seeded in 6-well plates (160 × 10^3^ or 130 ×10^3^ per well) and incubated for 48 h with JQ1 or N-JQ1 0.05 µM. For the adhesion assay, 5 × 10^4^ cells were plated into 24-well plates coated with collagen I(BD Biosciences, Milan, Italy) and after 1 h of incubation, cells were stained with 0.1% Crystal Violet solution, solubilized in 10% acetic acid. Cell attachment was quantified by measurement of absorbance at 560 nm with a Thermo Fisher Multiskan FC spectrophotometer, Thermo Fisher Scientific Inc., Waltham, MA, USA).

For the migration assay, 60 × 10^3^ cells, suspended in serum-free medium containing 1% BSA, were plated in the upper chamber of the transwell inserts with 8 μm pores (Costar, Euroclone, Milan, Italy). The lower chamber contained 600 µL of medium and 10% FBS as a chemotactic agent. After 6 h of incubation with JQ1 or N-JQ1, migrated cells were counted using a microscope (magnification 10×) provided with an eyepiece and equipped with a counting grid. Images of migrated cells were captured by microscope (Leica BM5500 B), equipped with a 20× objective.

In all experiments, cells treated with JQ1 vehicle or empty nanoparticles were used as controls. Results are expressed as percentages over the control.

### 4.4. Xenograft Model Development and Treatments

Five-week-old female athymic nude mice (*n* = 20) (Envigo, Huntingdon, UK) were maintained under specific pathogen-free conditions in sterile, ventilated microisolator cages at a constant temperature (24–26 °C), constant humidity (30%–50%) and a 12 h light/dark cycle. Sterilized food and tap water were given ad libitum. A total of 5 × 10^6^ MDA-MB 231 cells in 0.1 mL of Matrigel (BD Biosciences) were injected into intrascapular region of the mice. Treatment was initiated when the tumors reached an average volume of ~150 mm^3^. The mice were then randomly divided into the following four groups: animals treated with PBS + 5% PEG 400 + 5% TWEEN 80 (control), animals treated with JQ1 20 mg/Kg diluted in PBS + 5% PEG 400 + 5% TWEEN 80 (JQ1), animals treated with an aqueous suspension made up of unloaded nanoparticles (N-empty) or nanoparticles containing JQ1 20 mg/Kg (N-JQ1). The treatment was carried out for 5 days a week for two weeks by i.p. injection. Tumor development was followed at 2-day intervals by caliper measurements along two orthogonal axes: length (L) and width (W). The volume (V) of tumors was estimated by the following formula: V = L (W^2^)/2 [48]. Body weight, feeding behavior and motor activity of the mice were used as indicators of general health. At the end of each experiment, animals were euthanized by cervical dislocation after anesthesia with Avertin (2, 2, 2-Tribromoethanol, 250 mg/kg, i.p.), and the xenografts were harvested and subjected to downstream end-point analysis. Tumor tissues were excised, weighed and stored in formalin 10% for immunohistochemistry. All animals were maintained and handled in accordance with the recommendation of the Guidelines for the Care and Use of Laboratory Animals and experiments were approved by the Animal Care Committee of University of Calabria (OPBA), Italy (ethic code: 533/2019-PR, approved on 19 July 2019).

### 4.5. Histopathological Study and Immunohistochemistry

Tumor tissues were fixed in 10% formalinand subsequently processed with an automated tissue processor (Leica ASP 6025) and paraffin embedded. Sections (4 µm-thick) were mounted on coated glass slides, and heated at 60 °C for 60 min. One section was stained with hematoxylin and eosin for light microscopy analysis, while other sections were prepared for immunohistochemical analyses for which standard protocol was applied [49]. Tissue sections for immunohistochemical staining was performed with an automated immunostainer (Bond RX Max, Leica Biosystems, Buccinasco, Milan, Italy) using anti-CD 31 (ready to use, clone JC70A, Dako Agilent, Santa Clara, CA, USA) and anti-Ki67 (1:500) (ab6526, Abcam, Cambridge, UK) antibodies. A direct count of positive cells was conducted on ten fields for all the cases. The sections were stained with DAB and then slightly counterstained with Mayer hematoxylin, to be analyzed using a microscope (Leica LDM108) and digital image-capture computer system. Two senior pathologists (ChM and SC) analyzed the immunohistochemical results independently, blind to treatment, discriminating positively (brown color) and negatively (blue color) stained cells through manual counting. The results are expressed as percentage of positive cells.

### 4.6. RNA Extraction and Real Time PCR

The Trizol method (Thermo Fisher Scientific, Inc.) was used to extract total RNA from tumoral tissues. c-Myc gene levels were determined with real-time-PCR, using Platinum Sybr Green QPCR supermix and the ABI Prism 7300 Sequence Detection Systems (Applied Biosystems, Foster City, CA, USA), as previously described [12]. The *β-actin* gene was used as an endogenous reference. All amplification reactions were performed in triplicate, and the threshold cycles (identified with Applied Biosystems, SDS software) of the three reactions were averaged. Results were obtained by the 2^−ΔΔCt^ method. Oligonucleotide primers were purchased from Sigma-Aldrich, and their sequences are available upon request.

### 4.7. Statistical Analysis

Results were analyzed by one-way ANOVA followed by the Tukey–Kramer multiple comparisons test or Student’s *t*-test. All results are expressed as mean ± standard deviation (SD) and *p* values lower than 0.05 were considered statistically significant. All statistical analyses were performed using GraphPad Prism version 5.0 statistical software (GraphPad Software Inc., San Diego, CA, USA).

## 5. Conclusions

The present findings demonstrate that the encapsulation of JQ1 in nanoparticles, by protecting this compound and assuring a more efficient targeting to tumor cells, improves the anticancer efficacy of this innovative formulation against preclinical models of TNBC in vitro and in vivo, opening the way for a potential use in the treatment of TNBC.

## Figures and Tables

**Table 1:** Physico-chemical properties of poly-lactid-co-glycolic acid (PLGA) nanosystems loaded with JQ1.

JQ1 (mg/mL)	Mean Sizes (nm)	Polydispersity Index	Zeta Potential (mV)
−	130 ± 1	0.134 ± 0.01	−24 ± 1
0.1	145 ± 3 *	0.142 ± 0.04	−23 ± 2
0.25	148 ± 2 *	0.157 ± 0.05	−23 ± 1
0.5	158 ± 2 *	0.171 ± 0.04	−18 ± 2
1	275 ± 12 **	0.335 ± 0.15	−16 ± 2

* *p* < 0.05; ** *p* < 0.01 vs. empty formulation.
